# Post-COVID-19 Muscle Weakness and Recovery Patterns After Mild-to-Moderate Infection: A Retrospective Analysis of a Structured Rehabilitation Program Using the MRC Scale

**DOI:** 10.3390/healthcare14030392

**Published:** 2026-02-04

**Authors:** Ovidiu Cristian Chiriac, Daniela Miricescu, Raluca Mititelu, Silviu Marcel Stanciu, Corina Sporea, Ana Raluca Mitrea, Dragos Constantin Lunca, Sarah Adriana Nica, Cristian Constantin Popa, Ileana Adela Vacaroiu

**Affiliations:** 1 Discipline of Balneophysiokinetotherapy and Rehabilitation, Department of Specific Disciplines, Faculty of Midwifery and Nursing, Carol Davila University of Medicine and Pharmacy, 050474 Bucharest, Romania; ovidiu-cristian.chiriac@drd.umfcd.ro; 2 Rehabilitation Department, Central Military Emergency University Hospital “Dr. Carol Davila”, 010825 Bucharest, Romania; 3 Discipline of Biochemistry, Faculty of Dentistry, Carol Davila University of Medicine and Pharmacy, 050474 Bucharest, Romania; 4 Department of Nuclear Medicine, Carol Davila University of Medicine and Pharmacy, 050474 Bucharest, Romania; raluca.mititelu@umfcd.ro; 5 Nuclear Medicine Department, Central Military Emergency University Hospital “Dr. Carol Davila”, 010825 Bucharest, Romania; 6 Department of Internal Medicine and Gastroenterology, Carol Davila University of Medicine and Pharmacy, Central Military Emergency University Hospital “Dr. Carol Davila”, 010825 Bucharest, Romania; 7 Scientific Research Core, National University Center for Children’s Neurorehabilitation “Robănescu-Pădure”, 44 Dumitru Minca Street, 041408 Bucharest, Romania; 8 Physical Medicine and Rehabilitation (Medical Recovery Neurology), Carol Davila University of Medicine and Pharmacy, 050474 Bucharest, Romania; 9 Discipline of Pharmacology, Clinical Pharmacology, and Pharmacotherapy, Carol Davila University of Medicine and Pharmacy, 050474 Bucharest, Romania; 10 Department of Physical Medicine and Rehabilitation, Carol Davila University of Medicine and Pharmacy, 050474 Bucharest, Romania; 11 Department of General Surgery, Faculty of Medicine, Carol Davila University of Medicine and Pharmacy, 050474 Bucharest, Romania; cristian.popa@umfcd.ro; 12 General Surgery Department, University Emergency Hospital of Bucharest, 050098 Bucharest, Romania; 13 Department of Nephrology, Faculty of Medicine, Carol Davila University of Medicine and Pharmacy, 050474 Bucharest, Romania; ileana.vacaroiu@umfcd.ro

**Keywords:** COVID-19, structured rehabilitation, muscle strength, physical therapy, MRC scale, post-viral recovery

## Abstract

**Background/Objectives**: Post-COVID-19 muscle weakness is common even after mild or moderate infection, driven by systemic inflammation, prolonged inactivity, and reduced functional reserve. This study aimed to describe changes in global muscle strength assessed using the Medical Research Council (MRC) scale in adults recovering from mild or moderate COVID-19 who participated in a structured two-week rehabilitation program, and to compare these changes with those observed under standard medical follow-up. **Methods**: This retrospective study included 193 adults recovering from mild or moderate COVID-19: 160 who completed a structured inpatient rehabilitation program (study group) and 33 who received no supervised rehabilitation (control group). Muscle strength was assessed using the MRC scale at baseline and at follow-up. Non-parametric analyses (Wilcoxon signed-rank test, Mann–Whitney U test, and Spearman correlation) were used to evaluate within-group changes, between-group differences, and associations with age and sex. **Results**: Both groups showed significant within-group improvements in muscle strength. In the study group, median MRC scores increased from 50 (IQR 40–56) to 52 (IQR 50–56), with a mean ΔMRC of 2.76 ± 8.72 (*p* < 0.001). In the control group, MRC scores rose from 50 (40–56) to 52 (43–56), corresponding to a mean ΔMRC of 1.00 ± 2.09 (*p* = 0.005). The between-group comparison of ΔMRC did not reach statistical significance overall; however, age-stratified analyses indicated greater muscle strength gains in the rehabilitation group among participants aged ≥60 years. **Conclusions**: Short-term improvements in global muscle strength were observed both after structured rehabilitation and under standard medical follow-up, indicating a substantial contribution of natural recovery. Although participants in the rehabilitation group showed numerically larger gains—most notably in the ≥60-year subgroup—between-group differences in ΔMRC were not statistically significant. Overall, these findings support the feasibility and potential functional value of early, individualized rehabilitation while underscoring the need for adequately powered prospective studies to clarify its incremental benefit beyond spontaneous recovery.

## 1. Introduction

The SARS-CoV-2 infection has posed a major global health challenge, with more than 6.6 million deaths worldwide [1]. During the acute phase, the virus triggers both innate and adaptive immune responses, leading to uncontrolled inflammation and multisystem tissue injury [2]. SARS-CoV-2 enters the host through respiratory droplets, binding to angiotensin-converting enzyme 2 (ACE2) receptors on alveolar type II epithelial cells, which serve as the main viral entry point. This interaction promotes a cytokine storm, endothelial dysfunction, and diffuse alveolar damage [3]. In severe cases, extensive alveolar injury and microthrombosis contribute to complications such as pulmonary embolism and ischemic stroke [4,5]. These mechanisms can result in long-term pulmonary sequelae, including interstitial fibrosis and persistent respiratory limitation.

Severe COVID-19 outcomes are more frequently associated with older age, male sex, and pre-existing comorbidities such as obesity, diabetes, and cardiovascular disease [6,7]. Although most patients experience mild to moderate symptoms, approximately 10–20% develop acute respiratory distress syndrome requiring mechanical ventilation or prolonged bed rest [8,9]. The cumulative impact of systemic inflammation, hypoxemia, corticosteroid exposure, and inactivity leads to profound muscle atrophy and neuromuscular dysfunction [10].

Recent studies have highlighted that muscle wasting can begin after as little as 48 h of inactivity [10,11]. Prolonged immobilization impairs glucose metabolism, reduces muscle insulin sensitivity, and promotes oxidative stress [12,13]. Moreover, SARS-CoV-2 directly affects skeletal muscle fibers through mitochondrial dysfunction and altered renin–angiotensin signaling, resulting in decreased contractility and endurance [14,15]. These changes are often amplified in patients with critical illness or those who underwent invasive ventilation, where up to 75% develop intensive-care-unit-acquired weakness [16].

Beyond the acute stage, many patients continue to experience fatigue, dyspnea, and muscle weakness for months, a constellation of symptoms now recognized as part of the post-acute sequelae of COVID-19, or “long COVID” [17,18]. Persistent physical impairment is frequently associated with inflammatory and metabolic dysregulation [15,19], with functional limitations that may last up to one year after hospital discharge [20]. This ongoing muscle dysfunction significantly impacts quality of life and independence in daily activities [9,21].

Importantly, muscle weakness and reduced physical performance are not exclusive to older adults or critically ill patients. Worldwide, physical inactivity and aging represent major public health challenges, with declines in muscle strength and mass increasingly observed across all adult age groups, including younger individuals with sedentary lifestyles or excess caloric intake [22]. Obesity has also been shown to negatively affect maximal muscle strength—whether isotonic, isometric, or isokinetic—across the lifespan, from adolescence to older adulthood [23].

The loss of skeletal muscle mass in post-COVID-19 individuals is multifactorial. It reflects not only inactivity and systemic inflammation but also alterations in protein synthesis pathways, such as ubiquitin–proteasome and autophagy dysregulation [14]. In elderly patients, this process overlaps with age-related sarcopenia, increasing the risk of frailty and falls [15]. Conversely, physically active individuals display greater resilience to viral infections and faster functional recovery due to enhanced immune regulation and preserved mitochondrial function [12,24].

Skeletal muscle strength—defined as the maximal force generated by a muscle during a single effort—and muscle power, reflecting the ability to generate force rapidly, decline progressively with age and are strongly associated with poor quality of life, increased morbidity, disability, and mortality [25]. Consequently, muscle strength is now recognized not only as a determinant of physical function, but also as a predictor of chronic disease and overall health status [26].

Generally, as they age, older adults become more vulnerable to falls, hospitalizations, comorbidities, functional decline, and death. Additionally, muscle weakness driven by physiological changes in the musculoskeletal system represents a key factor increasing susceptibility to these adverse outcomes [27]. The age-related decline in skeletal muscle strength, muscle mass, and physical performance—commonly referred to as sarcopenia—remains largely underdiagnosed [28]. Low muscle mass and malnutrition are not limited to older adults and can occur at any age following acute or chronic illness, including COVID-19 [29].

Physical inactivity related to quarantine measures and hospitalization further accelerates muscle mass and strength loss, while inadequate nutritional intake during acute SARS-CoV-2 infection may exacerbate catabolic processes during recovery. Previous studies have reported malnutrition rates of approximately 42% among hospitalized COVID-19 patients, increasing to nearly 67% in those requiring intensive care. Reduced food intake due to gastrointestinal symptoms, anosmia, dysgeusia, anorexia, and increased metabolic demands represent major contributors to this phenomenon [15]. Even short periods of complete bed rest—up to 10 days—have been associated with marked reductions in muscle strength and power, including knee extension, knee flexion, and stair-climbing capacity, particularly in adults aged 60–85 years [30].

Muscle quality is influenced by both micro- and macroscopic changes in muscle structure and composition. Therefore, acute systemic conditions such as COVID-19 act as potent catabolic stimuli for skeletal muscle. The extent of muscle mass and functional loss depends on multiple factors, including pre-existing conditions (age, frailty, comorbidities), anorexia, severity of the inflammatory response, inadequate protein intake, and physical inactivity during the active phase of infection [31].

Skeletal muscle-related symptoms are common in both acute COVID-19 and post-acute sequelae of COVID-19 (PASC). Myalgia is frequently reported, with an incidence ranging from 19% to 33%, often accompanied by muscle weakness and elevated creatine phosphokinase levels, particularly in patients with PASC or prior ICU admission [14,32]. Beyond symptom burden, a large meta-analysis including over 1.9 million participants demonstrated that higher muscle strength is associated with reduced all-cause mortality and lower risk of cardiovascular disease, type 2 diabetes, metabolic syndrome, and cognitive decline, underscoring the clinical relevance of preserving and restoring muscle strength across populations [33]. Rehabilitation therefore represents a cornerstone of post-COVID-19 management. Early mobilization and progressive exercise have been shown to restore muscle strength, reduce anxiety and depressive symptoms, and improve pulmonary and cardiovascular capacity [21,34,35]. Structured physiotherapy, respiratory training, and occupational therapy can accelerate return to functional independence, even in elderly or comorbid patients [36]. A growing body of evidence supports integrative rehabilitation models combining physical exercise, nutritional support, and psychological interventions to optimize outcomes [21,37].

Despite the growing evidence supporting physical rehabilitation after COVID-19, there remains a lack of standardized criteria to objectively quantify recovery at the muscular level. Many studies have focused primarily on respiratory function, walking endurance, or patient-reported fatigue, while neglecting a direct assessment of global motor strength. Yet, muscle strength is among the most sensitive indicators of functional status and independence, and its improvement often precedes measurable gains in mobility or endurance [9,14].

The Medical Research Council (MRC) scale, initially developed for neuromuscular evaluation, provides a simple, reliable, and reproducible bedside method to assess muscle strength across multiple limb segments [10]. It has been validated in various rehabilitation settings, including post-ICU, neurological, and respiratory recovery programs [38,39,40,41]. By integrating MRC scoring into post-COVID-19 evaluation, clinicians can detect subtle but clinically meaningful changes in strength that may not be captured by endurance tests such as the six-minute walk test or the Berg Balance Scale.

Therefore, the aim of this retrospective study was to describe the evolution of global muscle strength, assessed using the Medical Research Council (MRC) scale, in adults recovering from mild or moderate COVID-19 who participated in a structured rehabilitation program, and to compare these changes with those observed under standard medical follow-up. Secondary objectives included exploratory analyses of age- and sex-related differences in recovery patterns. By providing objective data on strength evolution, this study contributes to defining measurable benchmarks for post-COVID-19 functional rehabilitation and supports the integration of systematic MRC monitoring into early recovery programs.

## 2. Materials and Methods

### 2.1. Study Design and Setting

This study was designed as a retrospective observational cohort analysis. Adult patients with a confirmed history of mild or moderate COVID-19 were evaluated in the Rehabilitation Department of the Central Military Emergency University Hospital “Dr. Carol Davila”, Bucharest, Romania.

A total of 193 patients met the inclusion criteria and were eligible for analysis. Of these, 160 patients participated in a structured physical rehabilitation program (rehabilitation group), while 33 patients received standard medical follow-up without supervised rehabilitation (control group).

For the purposes of the present retrospective analysis, only patients who completed the full structured rehabilitation program, including the supervised inpatient phase and the planned follow-up assessment, were included in the rehabilitation group. Patients who initiated but did not complete the structured program were not retained in the final analytical cohort, in order to ensure consistency of exposure and outcome assessment.

### 2.2. Age Stratification and Subgroup Definitions

Age stratification was performed using a cutoff of 60 years (<60 vs. ≥60 years). This threshold was selected based on its frequent use in COVID-19–related epidemiological and rehabilitation research, where individuals aged 60 years and older have been consistently reported to exhibit increased vulnerability to post-infectious functional decline, muscle weakness, and delayed recovery, even following mild or moderate disease [42].

International epidemiological analyses and public health frameworks commonly identify 60 years as a clinically meaningful boundary for defining older adult populations in the context of COVID-19 outcomes, including functional impairment, reduced physiological reserve, and increased rehabilitation needs [43]. In particular, large population-based studies have shown that functional limitations and prolonged post-COVID-19 symptoms increase markedly from the sixth decade of life onward, independent of acute disease severity [44].

This cutoff has also been adopted in post-COVID-19 rehabilitation studies to capture early aging-related vulnerability, reflecting age-associated physiological changes, increased comorbidity burden, and reduced neuromuscular adaptability, while remaining sensitive to functional decline that may occur prior to the conventional geriatric threshold of 65 years.

### 2.3. Participants and Eligibility Criteria

Eligible participants were adults (≥18 years) with a documented history of mild or moderate COVID-19, classified according to internationally accepted criteria. Mild cases presented with constitutional or upper respiratory symptoms without hypoxemia or radiological pneumonia, while moderate cases exhibited respiratory symptoms with peripheral oxygen saturation <94% or required closer clinical monitoring.

Although the present cohort included exclusively patients with mild or moderate COVID-19, post-infectious muscle weakness has been consistently described across the entire severity spectrum, including non-ICU populations. Previous studies in critically ill patients have demonstrated that prolonged inactivity and systemic inflammation lead to rapid loss of muscle mass and strength, even in the absence of direct neuromuscular injury. These mechanisms are not exclusive to ICU settings and may also occur, to a lesser extent, in hospitalized or home-isolated patients with mild or moderate disease [45,46].

Patients were excluded if they had conditions that could independently affect muscle strength assessment or rehabilitation response, including chronic heart failure, chronic respiratory insufficiency, advanced neurological disorders, or severe pre-existing musculoskeletal pathology.

Controlled comorbidities such as arterial hypertension, dyslipidemia, or type 2 diabetes mellitus were present in a proportion of participants but did not contraindicate participation in rehabilitation. Detailed stratification by comorbid burden, body mass index, physical activity level prior to COVID-19, duration of hospitalization, ICU admission, or use of mechanical ventilation was not feasible due to incomplete retrospective documentation and was therefore not included in the statistical analysis.

### 2.4. Group Allocation (Rehabilitation vs. Control)

Group allocation was determined by routine clinical pathways and institutional logistics rather than by study-related criteria. Patients included in the rehabilitation group were referred to the structured recovery program based on functional impairment, clinical stability, and availability of rehabilitation services at the time of evaluation.

The control group consisted of patients who were managed under standard medical care without participation in a structured, supervised rehabilitation program. These patients did not receive a standardized physiotherapy protocol, scheduled rehabilitation sessions, or progression-based intervention comparable to that of the rehabilitation group.

As part of routine care, some control-group patients performed independent mobilization and basic verticalization, occasionally assisted by nursing staff, and received general lifestyle and activity advice in accordance with institutional practice. These measures were not supervised by rehabilitation specialists, not protocolized, and not systematically recorded as rehabilitation exposure.

No patient was denied indicated rehabilitation for study purposes, and all patients remained free to access rehabilitation services if their clinical condition or functional status changed.

### 2.5. Timing of Assessments

Baseline muscle strength assessment was performed at the time of post-COVID-19 functional evaluation for all participants. Follow-up MRC assessment was conducted approximately two weeks later in the rehabilitation group, corresponding to completion of the structured program. In the control group, the follow-up MRC assessment was extracted from routine clinical re-evaluation performed at approximately the same 2-week interval after baseline assessment.

### 2.6. Outcome Measures

#### 2.6.1. Medical Research Council (MRC) Scale

Global muscle strength was evaluated bilaterally using the Medical Research Council (MRC) scale. The following muscle groups were assessed:

- Upper limbs: shoulder abduction, elbow flexion, wrist extension;
- Lower limbs: hip flexion, knee extension, ankle dorsiflexion.

Each muscle group was graded on a 6-point ordinal scale (0–5), where:

- 0 = no visible or palpable muscle contraction;
- 1 = flicker or trace of contraction;
- 2 = active movement with gravity eliminated;
- 3 = active movement against gravity;
- 4 = active movement against gravity and resistance;
- 5 = normal muscle strength.

The total MRC score was calculated as the sum of individual muscle scores, yielding a global measure of muscle strength. When direct testing of a specific muscle group was limited by joint discomfort or localized impairment, contralateral or adjacent muscle groups were assessed and results extrapolated in accordance with standard clinical practice.

The primary outcome was the change in muscle strength (ΔMRC), calculated as the absolute difference between follow-up and baseline total MRC scores (ΔMRC = MRC_final − MRC_initial). Percentage change (ΔMRC%) was not calculated, as baseline MRC values were relatively high and heterogeneous, and absolute change was considered more appropriate for describing short-term functional evolution in this non-ICU population.

#### 2.6.2. Clinical and Functional Classification

Functional status was documented using a structured clinical-functional classification aligned with the International Classification of Functioning, Disability and Health (ICF). This classification is routinely used in the institutional rehabilitation pathway to guide therapeutic decision-making and progression, based on level of cooperation, global muscle strength, balance capacity, and transfer ability, rather than disease severity alone.

The classification comprises six functional levels (0–5):

- Level 0—Non-cooperative, non-evaluable patients

Patients unable to meet minimal evaluation criteria and fully non-cooperative (S5Q level 0).

Management focuses on postural care of trunk and limbs, regular repositioning at least every two hours, pressure sore prevention, and the use of orthoses to prevent maladaptive postures.

- Level 1—Non-cooperative or partially cooperative patients

Patients with limited or absent cooperation (S5Q levels 1–5), for whom transfers from supine to sitting or standing are contraindicated.

Interventions include assisted positioning, orthotic support, promotion of Fowler position, passive and active-assisted joint mobilization, neuromuscular electrical stimulation, and bed-based cycle ergometry for upper and/or lower limbs.

- Level 2—Cooperative patients with limited functional capacity

Patients cooperative (S5Q levels 3–5) who meet minimal evaluation criteria but are unable to actively perform transfers.

Rehabilitation includes assisted sitting, passive or assisted verticalization using tilt tables or adjustable beds, joint mobilization, resistance training, passive to active cycle ergometry, and neuromuscular electrical stimulation.

- Level 3—Reduced functionality, partial cooperation:

Cooperative patients (S5Q level 4–5) with total MRC score >36 and limited balance and transfer capacity.

Rehabilitation focuses on assisted orthostatism, joint mobilization, progressive strengthening exercises, active cycle ergometry, initiation of standing training, neuromuscular electrical stimulation, and basic occupational therapy aimed at activities of daily living (ADLs).

- Level 4—Moderate functionality, full cooperation:

Fully cooperative patients (S5Q level 5) with total MRC score >48 and improved balance and transfer ability.

Interventions include active mobilization, resistance training, active cycle ergometry, supported gait training, neuromuscular electrical stimulation, and functionally oriented occupational therapy.

- Level 5—High functionality, full cooperation:

Preserved balance and mobility with minimal assistance.

Rehabilitation emphasized endurance training, advanced coordination exercises, independent or minimally assisted gait training, and reintegration into complex daily and instrumental activities.

Although the full classification framework (Levels 0–5) is presented for methodological transparency, only patients classified as Levels 3–5 were eligible for inclusion in the present analysis. Patients in Levels 0–2 were excluded because they were either non-cooperative, unable to undergo standardized strength assessment, or required purely supportive care without structured rehabilitation progression.

This classification was used exclusively to guide clinical management and exercise progression and was not employed as a primary outcome measure.

The applied clinical-functional classification was not intended as a validated outcome scale, but as a pragmatic framework to guide rehabilitation intensity and progression, in accordance with the International Classification of Functioning, Disability and Health (ICF). Similar functional stratification approaches have been widely used in early rehabilitation settings, including post-ICU recovery and post-acute respiratory conditions, where functional capacity, cooperation, and balance determine therapeutic pathways rather than disease severity alone [45,47].

### 2.7. Rehabilitation Program

The rehabilitation program was designed to address post-COVID-19 deconditioning through a progressive, structured approach, combining respiratory retraining, mobilization, muscle strengthening, postural control, balance re-education, and functional task retraining.

The intensity and progression of exercises were individualized according to clinical stability, oxygen saturation, heart rate response, perceived exertion, and overall functional tolerance.

The intervention consisted of two consecutive phases: an in-hospital supervised phase followed by an outpatient continuation phase. A standardized five-stage progression model was applied, allowing gradual advancement from bed-level mobilization to dynamic balance and strengthening activities. An overview of the rehabilitation protocol is presented in Table 1.

#### 2.7.1. In-Hospital Phase

Following clinical stabilization after COVID-19 treatment, patients entered a supervised 10-day inpatient rehabilitation program. Sessions were conducted five days per week, with a duration of approximately 30–45 min per session, and focused on early mobilization and prevention of further deconditioning.

- Core components of the in-hospital phase included: diaphragmatic and thoracic expansion breathing exercises;
- frequent postural changes (supine, lateral, and prone positioning every 2 h);
- active or active-assisted mobility exercises for upper and lower limbs (approximately 10 repetitions per muscle group);
- supported transfer to sitting and standing positions;
- supervised initiation of short-distance gait training.

Progression from bed-level activities to upright posture and ambulation was guided by oxygen saturation, heart rate, perceived exertion, and overall functional tolerance, with continuous supervision by physical therapists.

#### 2.7.2. Outpatient Phase

After hospital discharge, patients resumed rehabilitation within 30 days, participating in outpatient sessions twice weekly, each lasting approximately 30 min. This phase aimed to consolidate functional gains and promote reintegration into daily activities.

Outpatient rehabilitation included:

- Multi-axial dynamic balance training using the Huber 360 Evolution platform (LPG^®^ Systems, Valence, France);
- Targeted strengthening of hip, knee, and ankle stabilizing muscle groups;
- Aerobic reconditioning adapted to symptoms and cardiovascular response;
- Coordination and cognitive tasks;
- Functional retraining focused on independence in activities of daily living.

A five-stage progression model was applied, moving from basic postural control to advanced endurance and coordination exercises. Exercise intensity was increased gradually based on hemodynamic stability, motor control, and safe execution of prior stages. One rehabilitation cycle consisted of 10 outpatient sessions, which could be repeated every three months for up to one year, depending on individual clinical needs.

#### 2.7.3. Program Objectives

Across both phases, the primary objectives of the rehabilitation program were to:

- Restore respiratory efficiency;
- Counteract muscle weakness and atrophy;
- Improve balance and gait safety;
- Rebuild endurance and functional capacity;
- Enhance autonomy and quality of life.

All rehabilitation sessions were conducted under the supervision of licensed physical therapists, with medical oversight provided by a physiatrist.

#### 2.7.4. Control Group

Participants in the control group did not undergo a structured, supervised rehabilitation. program comparable to that applied in the rehabilitation group. They did not participate in protocolized physiotherapy sessions, progression-based exercise programs, or outpatient rehabilitation using specialized equipment. As part of routine clinical care, control-group patients received standard medical follow-up, general lifestyle and physical activity recommendations, and routine monitoring. Some patients performed independent mobilization and basic verticalization, occasionally assisted by nursing staff, according to their tolerance and level of cooperation. These activities were not standardized, not supervised by rehabilitation specialists, and not systematically recorded as rehabilitation exposure.

### 2.8. Ethical Approval

The study protocol was approved by the Ethics Committee of the Central Military Emergency University Hospital “Dr. Carol Davila” (Approval No. 694/28 March 2024) and complied with the Declaration of Helsinki. All patients provided informed consent for the use of anonymized clinical data.

### 2.9. Statistical Analysis

All statistical analyses were performed using IBM SPSS Statistics (version 29), and graphical representations were created using Microsoft Excel/Word 2024. Quantitative variables were expressed as means ± standard deviations or as medians with interquartile ranges. Normality was assessed using the Shapiro–Wilk test.

The change in muscle strength (ΔMRC) was calculated as the absolute difference between follow-up and baseline MRC scores (ΔMRC = MRC_final − MRC_initial). This approach was selected to provide a direct and clinically interpretable measure of strength recovery. Although relative change (ΔMRC%) has been used in ICU populations with very low baseline strength [48], absolute ΔMRC was considered more appropriate for this cohort of mild-to-moderate COVID-19 patients with preserved baseline function. In addition, the ordinal and bounded nature of the MRC scale may limit the interpretability of percentage transformations [49,50]. As MRC and ΔMRC values were not normally distributed, non-parametric tests were applied throughout. Within-group comparisons were conducted using the Wilcoxon signed-rank test, while between-group comparisons were performed using the Mann–Whitney U test. Associations between age and MRC parameters were evaluated using Spearman’s rank correlation coefficient.

The significance level was set at α = 0.05.

## 3. Results

### 3.1. Patient Characteristics

A total of 193 patients with mild or moderate post-COVID-19 condition were included in the analysis, of whom 160 participated in the structured rehabilitation program (study group) and 33 received standard medical follow-up without rehabilitation (control group). The overall mean age was 58.79 ± 13.21 years, with a similar age distribution between the study (58.54 ± 13.30 years) and control groups (59.97 ± 12.92 years). In the total cohort, 52.8% of patients were younger than 60 years and 47.2% were aged 60 years or older. Age distribution was comparable across groups, with patients ≥60 years representing 46.2% in the study group and 51.5% in the control group.

Women were slightly more represented overall (59.6%), and the sex distribution remained balanced between subgroups (study group: 60.6% women; control group: 54.5% women). Men accounted for 39.4% in the study group and 45.5% in the control group. These similarities in demographic characteristics indicate that the two groups were broadly comparable at baseline (Table 2).

### 3.2. Evolution of Muscle Strength in the Entire Cohort

Muscle strength, evaluated using the MRC scale at baseline and at follow-up, improved in both the study and control groups.

In the study group, the median MRC score increased from 50 (IQR 40–56) at baseline to 52 (IQR 50–56) after the rehabilitation program. This change was statistically significant (*p* < 0.001; Wilcoxon signed-rank test), with a mean improvement of 2.76 ± 8.72 points (median = 2).

In the control group, a smaller but still significant improvement was observed, from 50 (40–56) to 52 (43–56) (*p* = 0.005). The mean increase in this group was 1.00 ± 2.09 points (median = 0), indicating only minimal spontaneous recovery over the same period.

When comparing the magnitude of change between groups, the difference in ΔMRC did not reach statistical significance (*p* = 0.278; Mann–Whitney U test). Although the study group showed a numerically greater improvement (median ΔMRC = 2 vs. 0), the variability within groups reduced the ability to detect a statistically significant difference.

**Figure 1:**
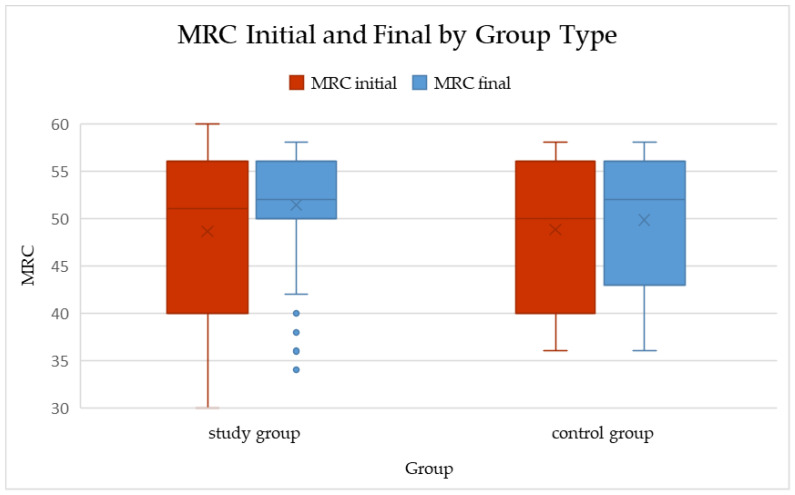
Boxplots of total MRC scores at baseline and follow-up in the study and control groups (median and IQR shown). Within-group changes: Study *p* < 0.001; Control *p* = 0.005 (Wilcoxon signed-rank). ΔMRC between groups: *p* = 0.278 (Mann–Whitney U).

Overall, these results suggest that while both natural recovery and rehabilitation may contribute to muscle strength improvement after COVID-19 infection, the present dataset did not demonstrate a statistically significant superiority of the structured rehabilitation program over standard follow-up (Table 3, Figure 1).

### 3.3. Sex-Based Differences in Muscle Strength Recovery

To evaluate whether sex influenced post-COVID-19 muscle strength recovery, MRC scores were analyzed separately for women and men within each study arm (Table 4). At baseline, women and men had comparable MRC values in both the study and control groups.

In the study group, both women and men demonstrated significant improvement following the two-week rehabilitation program. In the rehabilitation group, women improved significantly (*p* < 0.001), whereas men showed a borderline change (*p* = 0.079, not statistically significant).

In the study group, the magnitude of muscle strength improvement was comparable between women and men (median ΔMRC = 2 in both subgroups), with no statistically significant sex-related difference (*p* = 0.561). In the control group, changes were smaller: women showed no significant improvement (*p* = 0.084), while men exhibited a modest but statistically significant increase in MRC scores (*p* = 0.024). Overall, ΔMRC values in the control group remained low (median ΔMRC = 0), and no significant sex-related differences were observed (*p* = 0.435).

When ΔMRC values were compared between the rehabilitation and control groups within sex strata, no statistically significant differences were observed (*p* = 0.278), indicating that sex did not modify the association between rehabilitation exposure and muscle strength recovery.

Overall, these findings indicate that the rehabilitation program was similarly effective in women and men, with no evidence that sex influenced the capacity to regain muscle strength after mild or moderate COVID-19.

### 3.4. Age-Specific Differences

To investigate whether age influenced post-COVID-19 muscle strength recovery, participants were stratified into two subgroups: <60 years and ≥60 years, both within the study and control groups (Table 5).

In the study group, younger participants (<60 years) presented higher baseline MRC scores compared with older adults (≥60 years). Following the two-week rehabilitation program, both age subgroups showed an increase in muscle strength; however, statistical significance differed by age. In patients younger than 60 years, the median increase in MRC score was small and did not reach statistical significance (*p* = 0.257). In contrast, participants aged 60 years or older demonstrated a statistically significant and clinically meaningful improvement, with a median ΔMRC of 4 points (*p* < 0.001).

The between-group comparison confirmed that older adults improved significantly more than younger ones (*p* = 0.025). This suggests that individuals ≥60 years may derive comparatively greater benefit from structured rehabilitation in the post-COVID-19 phase.

In the control group, a similar age-related pattern was observed. Participants younger than 60 years did not exhibit a meaningful change in MRC scores over the follow-up period (*p* = 0.180). In contrast, individuals aged 60 years or older showed a modest but statistically significant improvement in muscle strength (*p* = 0.013).

The difference between the two age strata approached statistical significance (*p* = 0.050), suggesting that age may influence spontaneous recovery trajectories as well.

Study vs. control comparison

In age-stratified analyses, changes in muscle strength differed between the rehabilitation and control groups. Specifically, when ΔMRC was compared between the study and control groups within age strata, the difference reached statistical significance (*p* = 0.016), indicating a greater improvement in the rehabilitation group, particularly among participants aged ≥60 years.

Because age-stratified analyses revealed differential recovery patterns between the rehabilitation and control groups, we further explored whether a continuous association existed between age and muscle strength improvement.

### 3.5. Correlation Between Age and MRC Change

To further investigate the contribution of age to muscle strength recovery after COVID-19, Spearman’s rank correlation coefficients were calculated for the entire cohort and separately for the study and control groups.

**Figure 2:**
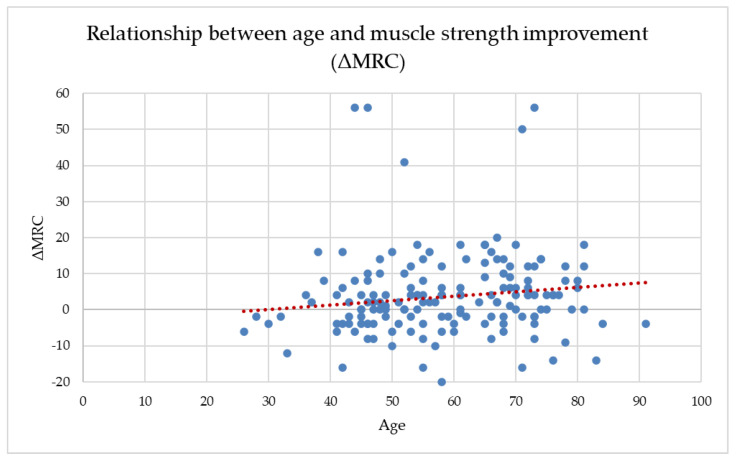
The relationship between age and MRC improvement (ΔMRC).

Across the full dataset, age showed a weak but statistically significant positive correlation with ΔMRC (r = 0.170, *p* = 0.020), suggesting that older participants tended to gain slightly more muscle strength during the follow-up period. Age also correlated negatively with both baseline and final MRC scores, indicating that older adults entered the program with lower muscle strength and maintained lower absolute values throughout recovery. This weak positive association is illustrated in Figure 2, where the wide dispersion of data points confirms the absence of a clinically meaningful age-dependent trend in ΔMRC.

When analyzed separately by group, age-related patterns differed between the rehabilitation and control cohorts. In the study group, age showed a weak but statistically significant positive correlation with ΔMRC (r = 0.172, *p* = 0.033), suggesting a tendency for older patients to experience slightly greater improvements in muscle strength following the structured two-week rehabilitation program. At the same time, age was negatively correlated with both baseline and final MRC scores, reflecting the expected age-related reduction in absolute muscle strength levels.

In the control group, no statistically significant association was observed between age and ΔMRC (r = 0.287, *p* = 0.106). Although older participants demonstrated marginally larger median improvements, the wide variability of responses and the limited sample size likely constrained the ability to detect a significant relationship, indicating that spontaneous recovery alone may result in less predictable strength gains.

These findings suggest that while age influences baseline functional status, it does not restrict the capacity for meaningful muscle strength recovery—particularly when patients participate in a structured rehabilitation program. In fact, older adults in the study group appeared to benefit comparably, and in some cases slightly more, than younger participants. This reinforces the value of tailored therapeutic exercise even in aging populations recovering from mild or moderate COVID-19 infection.

Overall, the results highlight a consistent improvement in muscle strength across age groups, with rehabilitation showing clear added value over natural recovery alone. The MRC scale proved to be a sensitive and clinically practical tool for quantifying these changes.

## 4. Discussion

Recovery after COVID-19 is often very important as more people contract the virus, making physical rehabilitation essential for restoring quality of life and supporting overall health. Physical training is known to improve cardiorespiratory capacity, muscle strength, and endurance—factors often impacted by coronavirus infection [51]. Aging is associated with changes across the social, mental, and physical aspects of life [52], and it also increases the risk of COVID-19 infection. Additionally, it makes individuals more likely to experience atypical presentations, severe disease forms, and higher mortality [53].

Skeletal muscle impairment is one of the most consistent functional consequences of SARS-CoV-2 infection, regardless of initial disease severity [9]. Reduced muscle mass and diminished strength arise from a complex interplay of systemic inflammation, mitochondrial dysfunction, and prolonged inactivity during illness and recovery [11,18]. The cytokine-driven hyperinflammatory response characteristic of COVID-19 promotes proteolysis through activation of the ubiquitin–proteasome pathway while suppressing anabolic IGF-1/AKT/mTOR signaling [10,14]. Additionally, dysregulation of the renin–angiotensin system increases oxidative stress and muscle catabolism [3], while impaired mitochondrial biogenesis further limits endurance and recovery capacity [15]. Together, these mechanisms explain the high prevalence of post-viral weakness, reduced exercise tolerance, and prolonged deconditioning reported after COVID-19 infection [6,17].

Periods of isolation and hospitalization have further amplified sedentary behavior, already recognized as a major global health concern [13,54]. Even brief reductions in daily activity can markedly decrease quadriceps thickness, neuromuscular efficiency, and metabolic function [14,16,55]. Rehabilitation in post-acute COVID-19 syndrome improves muscle strength, walking ability, sit-to-stand performance, and quality of life [56].

Muscle weakness is mainly observed in patients with more severe COVID-19. Physical activity is associated with reduced inflammation; therefore, individuals with better physical fitness might have some protection against COVID-19, since the most severe cases tend to occur in patients with higher inflammation levels [57].

Critically ill patients exhibit profound neuromuscular impairment—ICU-acquired weakness may affect more than 70% of ventilated individuals [15,16].

ICU patients with severe COVID-19 experienced a 30% reduction in the cross-sectional area of the rectus femoris and nearly a 20% decrease in the thickness of the anterior compartment of the quadriceps muscle after 10 days. Soares et al. reported that in 41 hospitalized patients (aged 40 to 88 years) recovering from acute COVID-19, knee extensor and arm flexor weakness were observed in 75–85% of patients [14]. The results conducted on 42 post-ICU COVID-19 patients showed that one month after rehabilitation, there was a significant improvement in limb and respiratory muscle strength, cough effectiveness, balance, exercise capacity, fatigue, and the ability to perform daily activities. Additionally, older age, longer hospital stays in acute care, depressive symptoms, and cognitive deficits were linked to poorer functional outcomes [58]. Muscle weakness was observed in 59% of post-hospitalized patients and in 65% of those with post-acute sequelae of COVID-19, approximately 14 weeks after their initial infection. Hospital stay duration and diabetes mellitus were identified as potential predictors of post-COVID-19 muscle weakness [59].

Furthermore, notable improvements in the MRC score were seen at 3 months in patients with post-acute COVID syndrome following a personalized rehabilitation program. Additionally, increases in respiratory muscle strength were linked to reductions in dyspnea and improved exercise capacity [60].

Accordingly, Rahiminezhad et al. reported that among 15 COVID-19 patients discharged from the ICU, arm and leg muscle strength—assessed using both a handheld dynamometer and the MRC scale—was significantly lower compared with non-COVID-19 ICU patients [61]. Similar findings were described by Taketa et al. in a cohort of 13 post-COVID-19 patients evaluated at ICU discharge using the MRC scale [62].

Lower MRC scores at discharge have been associated with older age [63] and with the greater severity of critical illness [64]. Moreover, patients aged ≥60 years or those requiring mechanical ventilation for more than 10 days experienced more pronounced muscle loss, reduced strength, and impaired mobility, as documented by Gutiérrez et al. [65].

However, accumulating evidence indicates that even mild and moderate COVID-19 can induce clinically relevant muscle dysfunction, primarily through inactivity, systemic inflammation, and transient hypoxemia [15,16].

The results from 28 nonhospitalized patients with post-COVID condition, who have no other diseases, show muscle changes such as smaller fiber size, lower phospholipid levels, reduced mitochondrial oxidative capacity, and decreased capillarization. These changes may result from lower physical activity levels compared to healthy subjects [66].

Due to the retrospective and non-randomized nature of the study, causal relationships between rehabilitation exposure and outcomes cannot be inferred. The present study adds to this body of evidence by demonstrating that both natural recovery and structured rehabilitation are associated with measurable short-term improvements in global muscle strength following mild or moderate COVID-19. Importantly, although the rehabilitation group exhibited numerically larger gains, the between-group comparison of ΔMRC did not reach statistical significance, underscoring the need for cautious interpretation.

In the study group, median MRC scores increased significantly after the two-week rehabilitation protocol, indicating that supervised mobilization, progressive strengthening, and respiratory exercises are feasible and beneficial in the early post-COVID-19 phase. Improvements of comparable magnitude have been reported in other post-COVID-19 cohorts undergoing structured recovery programs [34,37,67,68,69]. Romanian studies similarly documented improvements in dyspnea, effort tolerance, and cooperation following multidisciplinary post-COVID-19 rehabilitation [36], as well as reductions in anxiety and depressive symptoms that may indirectly facilitate physical recovery [21,70].

At the same time, the control group also demonstrated a small but statistically significant improvement, reflecting the contribution of spontaneous post-infectious recovery. The absence of a statistically significant difference in ΔMRC between groups may be explained by several factors, including the wide dispersion of MRC values, heterogeneity in the duration of symptoms prior to assessment, the relatively short follow-up interval, and the imbalance in group sizes. Similar attenuation of between-group effects due to early spontaneous recovery has been reported in other post-viral rehabilitation studies [34]. Comparable patterns have also been described in orthopedic and neurological rehabilitation, where natural recovery may coexist with therapy-related gains during early phases, complicating short-term effect detection [71].

### 4.1. Sex- and Age-Related Recovery Patterns

Despite this statistical limitation, several clinically relevant patterns emerged:

Muscle strength recovery was comparable between women and men, with no statistically significant sex-related differences in ΔMRC. Both sexes demonstrated similar responses to the rehabilitation program, consistent with previous reports showing that when exercise intensity and progression are individualized, recovery trajectories are largely independent of sex [15,72,73,74]. Comparable neuromuscular activation patterns and metabolic responsiveness to progressive loading may underlie this finding.

Age showed a more nuanced relationship with recovery. Younger participants (<60 years) exhibited only small, nonsignificant improvements, whereas adults aged ≥60 years demonstrated statistically significant and clinically meaningful gains. Moreover, within the rehabilitation group, age showed a weak but significant positive correlation with ΔMRC, suggesting that older adults may derive particular benefit from structured rehabilitation. These findings align with evidence indicating that appropriately dosed physical training can partially overcome age-related anabolic resistance and stimulate neuromuscular plasticity, even in older populations [19,71]. Other studies have similarly reported meaningful improvements in walking capacity, endurance, and functional independence among older adults enrolled in post-COVID-19 or tele-rehabilitation programs [75,76,77].

### 4.2. Physiological Considerations

Given the short duration of the intervention, the observed improvements in MRC scores are most plausibly explained by early neural and functional adaptations, rather than by substantial structural muscle remodeling. Repeated activation may enhance motor unit recruitment, coordination, and neuromuscular efficiency [78], while respiratory and postural exercises improve oxygen delivery and reduce hypoxemic stress [18]. Although aerobic and resistance training are known to increase mitochondrial density and oxidative capacity [14], such adaptations typically require longer training periods. Therefore, in the present context, improvements likely reflect functional reactivation and improved neuromuscular control rather than extensive mitochondrial or structural changes.

Comparable early adaptations have been described in other rehabilitation fields. In stroke survivors, short-term aerobic and resistance interventions primarily enhance functional capacity through improved neuromuscular efficiency before structural changes occur [79,80]. Similar mechanisms have been reported following musculoskeletal trauma, where progressive loading initially improves strength and coordination through neural adaptation [81], and in chronic neuromuscular disorders, where structured exercise reduces fatigue and improves functional performance [82,83].

### 4.3. Clinical Implications

Importantly, the magnitude of strength gains observed in the rehabilitation group should be interpreted in the context of the relatively short duration and moderate intensity of the intervention, which focused primarily on early mobilization, respiratory re-education, and progressive functional strengthening rather than high-load or long-term resistance training. Within this framework, the observed improvements in MRC scores likely reflect clinically meaningful early functional reactivation rather than maximal strength restoration. Although no statistically significant between-group differences were observed in sex-stratified analyses, age-stratified comparisons demonstrated greater muscle strength gains in the rehabilitation group, particularly among participants aged ≥60 years. This finding suggests that early, structured rehabilitation may confer additional functional benefits beyond spontaneous recovery in older individuals, who typically present with lower baseline muscle strength and reduced physiological reserve. At the same time, the presence of measurable improvement in the control group highlights the contribution of natural post-infectious recovery, emphasizing that rehabilitation should be viewed as a supportive and potentially accelerating intervention rather than a replacement for spontaneous functional restoration. A median improvement of 2–4 points on the MRC scale corresponds to meaningful functional gains, including improved ability to rise from a chair, initiate gait, climb stairs, and perform activities of daily living [24]. Enhanced limb and trunk strength may also support ventilatory mechanics and postural stability [37,41], reinforcing current recommendations for early mobilization after SARS-CoV-2 infection [84,85,86]. In addition to improvements in global muscle strength, structured post-COVID-19 rehabilitation has been shown to positively influence functional mobility and gait performance. Recent evidence demonstrates significant gains in TUGand 10-Meter Walk Test (10 MWT) outcomes following targeted rehabilitation programs in post-COVID-19 patients, highlighting the broader functional impact of early therapeutic intervention beyond isolated strength measures [87]. These findings support the clinical relevance of even modest MRC improvements, as gains in muscle strength often precede and facilitate measurable improvements in mobility, balance, and walking capacity.

The present findings are consistent with multimodal rehabilitation strategies applied in other clinical contexts, such as post-transplant recovery, adhesive capsulitis, knee ligament injury, and musculoskeletal trauma, where progressive exercise supports functional restoration [88,89,90,91,92]. In line with previous observations, individuals with mild-to-moderate COVID-19 often recover to pre-illness functional levels, whereas patients with severe disease experience slower and less complete recovery, contributing to a substantial socioeconomic burden [93].

Evidence from long COVID cohorts further supports the role of rehabilitation. Ostrowska et al. reported significant improvements in body composition and dyspnea following a six-week multidisciplinary program [94], while Tramonti et al. demonstrated improved exercise capacity, fatigue, dyspnea, and quality of life after a structured home-based pulmonary rehabilitation program [95]. Cross-sectional analyses also highlight persistent functional limitations and reduced quality of life among post-COVID-19 patients [96], underscoring the need for targeted rehabilitation strategies.

### 4.4. Strengths, Limitations, and Future Directions

Strengths of this study include the relatively large rehabilitation cohort, the use of a standardized MRC scoring protocol performed by trained physiotherapists, and detailed subgroup analyses stratified by age and sex. These features enhance internal consistency and facilitate comparison with existing literature.

Several limitations should be acknowledged. First, the retrospective and non-randomized design limits causal inference and introduces potential selection bias. Although improvements were observed in both groups, it cannot be concluded that rehabilitation was superior to natural recovery within this specific dataset. Second, the control group was considerably smaller than the rehabilitation group, which may have reduced statistical power and increased the risk of a type II error. Third, the short follow-up interval precludes conclusions regarding the long-term sustainability of observed gains.

Fourth, muscle strength was assessed exclusively using the MRC scale. While practical and widely used, the MRC is semi-quantitative and subject to ceiling effects, particularly in patients with mild impairment. The absence of complementary objective measures such as handgrip dynamometry or functional performance tests represents an additional limitation. In addition, due to the retrospective nature of the study and reliance on routine clinical documentation, detailed session-level data regarding rehabilitation adherence, missed sessions, and the proportional contribution of individual intervention components (e.g., respiratory training versus strengthening or balance exercises) were not systematically available. Consequently, analyses reflecting dose–response relationships or component-specific effects could not be performed and should be addressed in future prospective studies. Fifth, potentially relevant confounders—including comorbidities, nutritional status, prior physical activity levels, and psychosocial factors—were not analyzed. BMI was not available for adjustment in this retrospective dataset, although evidence from musculoskeletal rehabilitation suggests that higher BMI may influence functional recovery trajectories [97]. Future research should prioritize prospective, adequately powered studies with longer follow-up, integration of objective functional outcomes, and multimodal rehabilitation approaches addressing physical, respiratory, nutritional, and psychological domains. Psychosocial and behavioral factors that may affect adherence and recovery were not assessed [98]. These observations underscore the importance of integrating psychosocial and nutritional assessments into future post-COVID-19 rehabilitation studies to better capture the multidimensional determinants of functional recovery. Such designs will help clarify recovery trajectories and optimize rehabilitation strategies for post-COVID-19 populations.

## 5. Conclusions

This retrospective analysis shows that global muscle strength improved over a short follow-up period in adults recovering from mild or moderate COVID-19, both in patients who participated in a structured two-week rehabilitation program and in those receiving standard medical follow-up. These findings indicate that early post-infectious recovery is accompanied by measurable gains in muscle strength, as assessed using the Medical Research Council (MRC) scale.

Although patients enrolled in the rehabilitation program demonstrated numerically larger improvements, particularly among those aged ≥60 years, the between-group difference in ΔMRC did not reach statistical significance. Consequently, within the limits of this study design, the results do not support a definitive conclusion regarding the superiority of structured rehabilitation over natural recovery. Rather, they suggest that rehabilitation is associated with clinically relevant within-group improvements, especially in older adults who entered recovery with lower baseline strength.

Sex did not appear to influence recovery patterns, as women and men showed comparable changes in MRC scores. Across the entire cohort, age was only weakly correlated with strength improvement, indicating that chronological age alone does not preclude short-term neuromuscular recovery when patients are clinically stable and functionally active.

Taken together, these findings support the role of early, accessible, and individualized rehabilitation as a feasible strategy to counteract post-COVID-19 deconditioning, particularly in older adults, while acknowledging that spontaneous recovery also contributes substantially to early strength gains. The MRC scale proved to be a practical bedside tool for capturing short-term changes in global muscle strength in this population.

Future research should focus on prospective, adequately powered studies with balanced comparison groups, longer follow-up periods, and complementary objective functional outcomes to better characterize recovery trajectories and to define the specific contribution of rehabilitation interventions beyond natural post-infectious recovery. Multidisciplinary approaches integrating physical training, respiratory therapy, nutritional support, and psychosocial interventions are likely to be essential for optimizing long-term functional outcomes after COVID-19.

## Figures and Tables

**Table 1:** Structure of the post-COVID-19 rehabilitation program.

Phase/Setting	Main Objectives	Core Interventions	Frequency and Duration	Progression Criteria
Early in-hospital phase (Stage 1) (bed-level rehabilitation)	Prevent early deconditioning Improve respiratory efficiency Maintain joint mobility	Diaphragmatic and thoracoabdominal breathing exercises in supine, lateral, and prone positions Regular postural changes (every 2 h) Incentive spirometry (3 sessions/day) Active-assisted upper and lower limb movements (flexion, extension, abduction; ~10 repetitions per exercise)	Daily during hospitalization (multiple short sessions/day, 10 days)	Stable oxygen saturation Hemodynamic stability Ability to tolerate prolonged sitting
Mobilization and verticalization phase (Stage 2)	Improve trunk control Initiate upright posture Reduce orthostatic intolerance	Supported sitting at bedside Gradual verticalization with therapist assistance Continued respiratory training	Daily during hospitalization	Independent sitting Tolerance to upright posture without significant desaturation or hypotension
Gait initiation phase (Stage 3)	Restore standing balance Initiate ambulation	Supervised standing Short-distance walking (3–10 m), repeated several times/day Continued limb strengthening and breathing control	Daily during late inpatient phase	Independent standing Safe initiation of gait with or without minimal assistance
Outpatient rehabilitation—postural and balance training (Stage 4)	Improve balance and postural control Enhance coordination and endurance	Dynamic multi-axial balance training using the Huber 360 Evolution platform Postural stabilization exercises Aerobic reconditioning adapted to tolerance	2 sessions/week × ~30 min, 10 sessions per cycle	Hemodynamic stability during dynamic tasks Achievement of functional level compatible with independent standing and transfers
Outpatient rehabilitation—strengthening and functional integration (Stage 5)	Increase lower limb strength Improve gait efficiency Reinforce functional independence	Targeted strengthening of hip, knee, and ankle stabilizers Dynamic coordination tasks Functional exercises simulating activities of daily living (ADLs)	2 sessions/week × ~30 min 10 sessions per cycle (repeated every 3 months, up to 1 year if needed)	Functional autonomy (levels 4–5) Independent or minimally assisted ADLs
Overall program goals	Restore global muscle strength Improve respiratory capacity and endurance Enhance balance and coordination Promote independence and quality of life	—	—	—

**Table 2:** Baseline characteristics of the study population.

Variable	Age (Years), Mean ± SD	<60 Years, *n* (%)	≥60 Years, *n* (%)	Women, *n* (%)	Men, *n* (%)
Total (*n* = 193)	58.79 ± 13.21	102 (52.8%)	91 (47.2%)	115 (59.6%)	78 (40.4%)
Study group (*n* = 160)	58.54 ± 13.30	86 (53.8%)	74 (46.2%)	97 (60.6%)	63 (39.4%)
Control group (*n* = 33)	59.97 ± 12.92	16 (48.5%)	17 (51.5%)	18 (54.5%)	15 (45.5%)

**Table 3:** Evolution of MRC Score in the Study and Control Groups.

MRC Evaluation	Mean ± SD	Median (IQR)	*p*-Value
Study Group (*n* = 160)			
Initial	48.55 ± 8.69	50 (40–56)	<0.001 *
After recovery	51.31 ± 5.80	52 (50–56)	
Difference (ΔMRC)	2.76 ± 8.72	2 (−4–8)	
Control Group (*n* = 33)			
Initial	48.82 ± 7.79	50 (40–56)	0.005 *
After recovery	49.82 ± 7.25	52 (43–56)	
Difference (ΔMRC)	1.00 ± 2.09	0 (0–2)	
*p* (Δ control vs. study)			0.278 **

* Wilcoxon signed-rank test (within-group comparison). ** Mann–Whitney U test (between-group comparison).

**Table 4:** Comparison of MRC improvement by gender.

Group/Sex	MRC Initial Mean ± SD	MRC Final Mean ± SD	ΔMRC Mean ± SD	Median ΔMRC (IQR)	*p*-Value (Initial vs. Final)
Study Group (*n* = 160)					
Women (*n* = 97)	48.81 ± 7.84	51.65 ± 6.12	2.84 ± 7.30	2 (−2–6)	<0.001 *
Men (*n* = 63)	48.16 ± 9.89	50.81 ± 5.30	2.65 ± 10.57	2 (−4.5–12)	0.079 *
*p* (Women vs. Men)					0.561 **
Control Group (*n* = 33)					
Women (*n* = 18)	49.83 ± 7.89	49.47 ± 7.62	0.53 ± 0.92	0 (0–2)	0.084 *
Men (*n* = 15)	48.72 ± 7.94	50.11 ± 7.15	1.39 ± 2.68	0 (0–2.25)	0.024 *
*p* (Women vs. Men)					0.435 **
*p* (ΔMRC Study vs. Control)					0.278 **

* Wilcoxon signed-rank test (within-group comparison). ** Mann–Whitney U test (between-group comparison).

**Table 5:** Comparison of MRC improvement by age category and correlation with age.

Group/Age	MRC Initial Mean ± SD	MRC Final Mean ± SD	ΔMRC Mean ± SD	Median ΔMRC (IQR)	*p*-Value (Initial vs. Final)
Study Group (*n* = 160)					
<60 years (*n* = 86)	50.59 ± 9.02	52.02 ± 5.63	1.44 ± 8.80	0.5 (−4–4.5)	0.257 *
≥60 years (*n* = 74)	46.46 ± 7.60	50.49 ± 5.97	4.03 ± 8.30	4 (−2–11.5)	<0.001 *
*p* (<60 years vs. ≥60 years)					0.025 **
Control Group (*n* = 33)					
<60 years (*n* = 16)	53.63 ± 5.67	54.00 ± 5.51	0.37 ± 1.09	0	0.180 *
≥60 years (*n* = 17)	44.29 ± 6.82	45.88 ± 6.54	1.59 ± 2.62	2 (0–2)	0.013 *
*p* (<60 years vs. ≥60 years)					0.05 **
*p* (ΔMRC Study vs. Control)					0.016 **

* Wilcoxon signed-rank test (within-group comparison). ** Mann–Whitney U test (between-group comparison).

## Data Availability

The data presented in this study are available on request from the corresponding author. The data are not publicly available due to privacy and ethical restrictions. The data presented in this study are available on request from the corresponding author.
